# Modified bentall surgery using a graft insertion technique for prosthetic valve endocarditis with left ventricular–aortic discontinuity: a case report

**DOI:** 10.1186/s44215-026-00243-8

**Published:** 2026-02-12

**Authors:** Daichi Sakurahara, Eisaku Nakamura, Kosuke Mori, Koji Furukawa

**Affiliations:** 1 Department of Cardiovascular Surgery, Miyazaki Prefectural Miyazaki Hospital, Miyazaki, 5-30 Kitatakamatsucho, Miyazaki-city, 880-8510 Miyazaki Japan; 2 Department of Cardiovascular Surgery, Faculty of Medicine, University of Miyazaki, 5200 Kiyotakecho Kihara, Miyazaki, Miyazaki-city, 889- 1692 Miyazaki Japan

**Keywords:** Infective aortic valve endocarditis, Left ventricular–aortic discontinuity, Modified bentall surgery, Graft insertion technique

## Abstract

**Background:**

Infective prosthetic valve endocarditis is a life-threatening condition with high morbidity and mortality; it is often complicated by periannular abscesses and left ventricular–aortic (LV–Ao) discontinuity. These situations make standard valve or root replacement technically challenging and require complex reconstructive procedures.

**Case presentation:**

We report a 59-year-old man with a history of hemiarch replacement and aortic valve replacement for acute Stanford type A dissection. He presented with fever and heart failure symptoms. Imaging revealed severe prosthetic valve regurgitation, periannular abscess, pseudoaneurysm, and LV–Ao discontinuity. Urgent surgery was performed. After complete debridement, the LV outflow tract was reconstructed using the graft insertion technique (GIT) with a Gelweave Valsalva graft and composite graft replacement. The coronary arteries were reconstructed with short interposition grafts. Postoperatively, antibiotics were continued; a permanent pacemaker was implanted for complete atrioventricular block; and the patient was discharged on postoperative day 54. At 1-year follow-up, he remained free of recurrent infection.

**Conclusions:**

The GIT facilitates secure fixation, reliable hemostasis, and reproducible reconstruction even in fragile, infected tissue. Although potential risks, including conduction disturbance and graft infection remain, the method is technically straightforward and can be applied during emergencies. In the case presented here, this approach resulted in satisfactory early outcomes, highlighting the applicability of graft insertion for managing LV–Ao discontinuity in a patient with prosthetic valve endocarditis, particularly when preparation time and graft availability are limited.

## Background

Infective aortic valve endocarditis is a life-threatening condition with high morbidity and mortality. Periannular extension of infection and abscess formation have been reported in 9.8%–40% of cases, depending on cohort and definition [1, 2]. The incidence of prosthetic valve infection is higher than that of native valve infection [1], and perivalvular complications occur in approximately 60% of patients with prosthetic valve endocarditis [3]. Development of periannular abscesses and false aneurysms is associated with a more aggressive clinical course. Complete removal of infected tissue may lead to left ventricular–aortic (LV–Ao) discontinuity, which complicates valve or root replacement and requires complex reconstruction [4, 5]. Herein, we report a case in which a modified Bentall procedure using a graft insertion technique (GIT) was successfully performed, achieving favorable early outcomes.

### Case presentation

A 59-year-old man was referred to our hospital with fever and symptoms of heart failure. Three years earlier, he had undergone hemiarch replacement and aortic valve replacement for acute Stanford type A dissection. Two weeks before this presentation to our hospital, fever was noted and his physician in the local region diagnosed him with a urinary tract infection. The treatment did not resolve the fever and he was transported to his previous doctor due to worsening heart failure symptoms, before being urgently transferred to our hospital with suspected infective endocarditis.

**Fig. 1:**
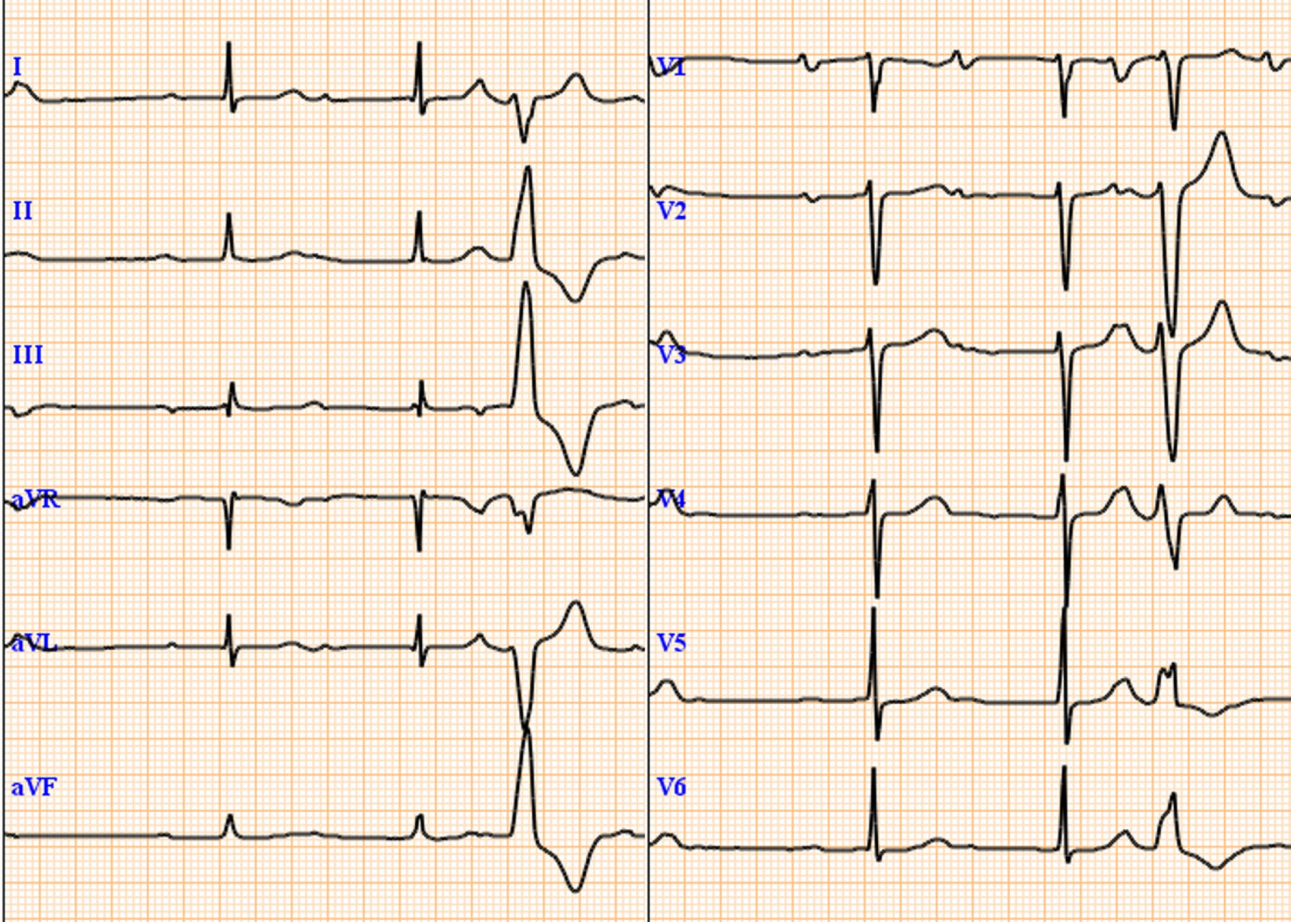
Electrocardiogram showing complete atrioventricular block Preoperative electrocardiography indicating complete atrioventricular block

**Fig. 2:**
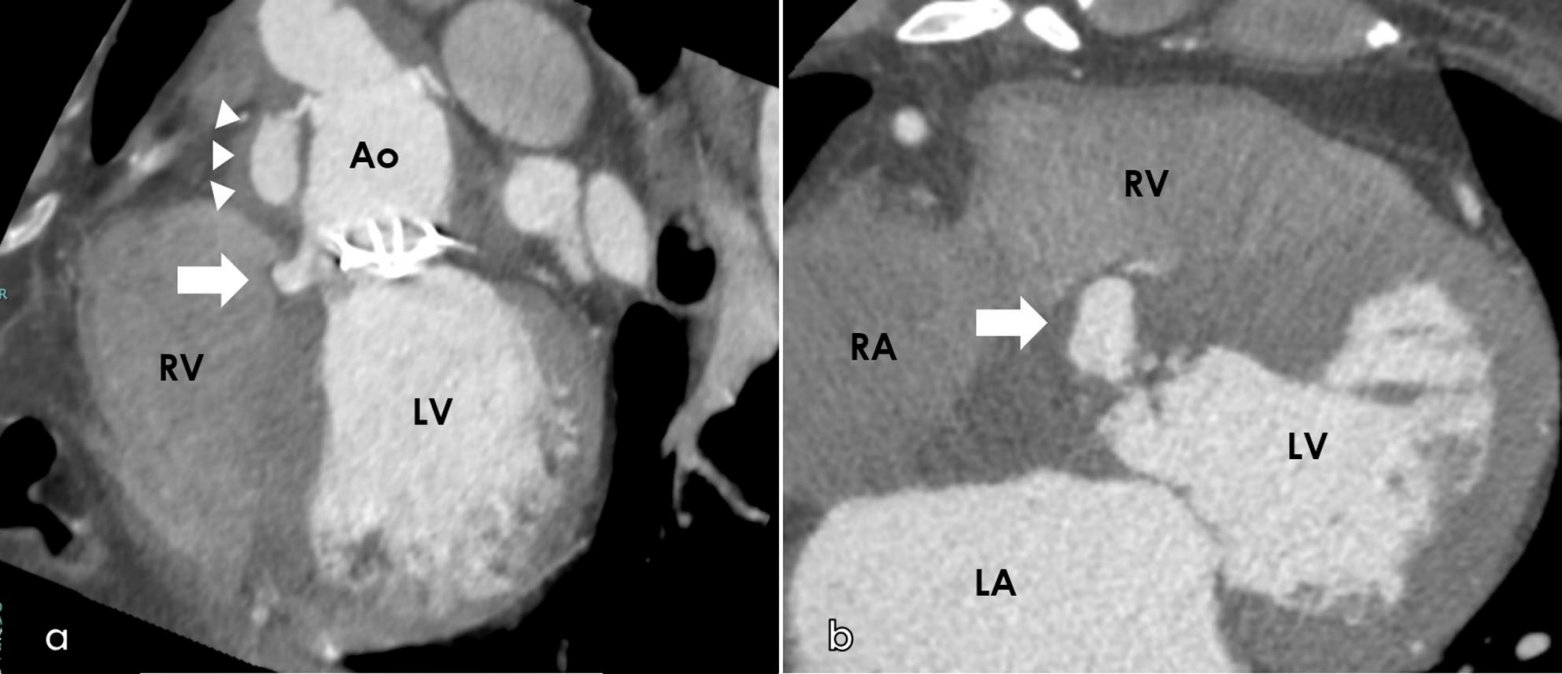
Cardiac computed tomography showing the formation of a periannular abscess extending into the ventricular septum (**a**) Long-axis view. A periannular cavity (white arrow) is visible at the level of the right coronary cusp of the aortic valve, extending deeply into the ventricular septum toward the right ventricle. A pseudoaneurysm is also observed at the proximal anastomosis site following hemiarch replacement (white arrowhead) (**b**) Short-axis view. The periannular abscess (white arrow) encroaches upon the ventricular septum, demonstrating its deep extension into the septal tissue *Ao* ascending aorta, *RA* right atrium, *LA* left atrium, *RV* right ventricle, *LV* left ventricle

**Fig. 3:**
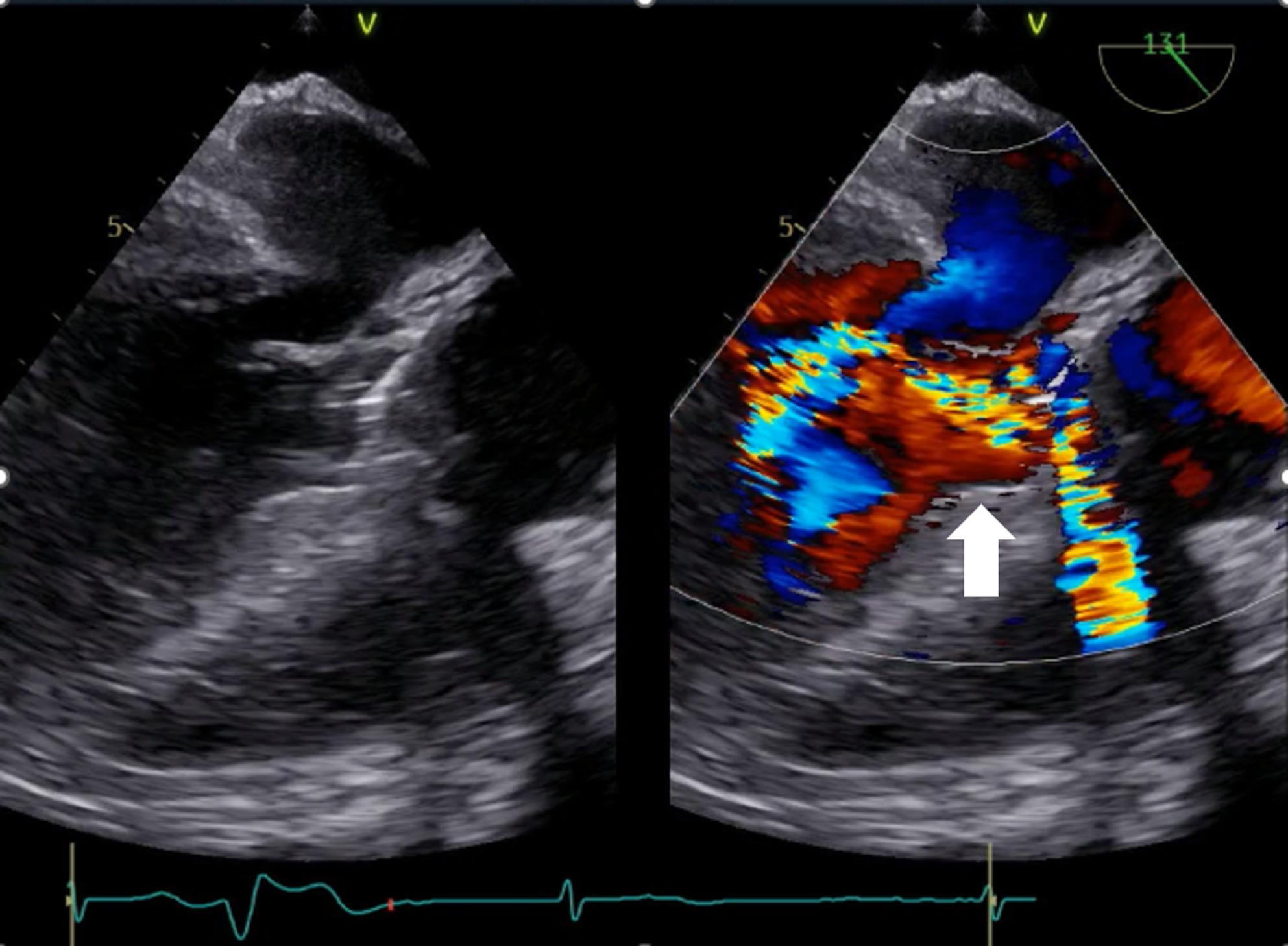
Transesophageal echocardiography showing paravalvular regurgitation surrounding the aortic valve prosthesis Preoperative transesophageal echocardiography revealing severe regurgitation around the prosthetic valve in the position of the aortic valve (white arrow)

On arrival, his blood pressure, heart rate, and body temperature were 132/70 mmHg, 61 bpm, and 35.8 °C, respectively. Oxygen saturation was 99% with oxygen delivered via a nasal cannula (2 L/min). A diastolic murmur was noted with its peak intensity at the left border of the third intercostal space. Blood test results revealed: white blood cells, 9320/µL; neutrophils, 7410/µL; red blood cells, 3.28 × 10^6^/µL; hemoglobin, 8.7 g/dL; hematocrit, 27.8%; platelets, 150 × 10^3^/µL; creatinine, 1.18 mg/dL; C-reactive protein, 5.40 mg/dL; NT-proBNP, 12,237 pg/mL; prothrombin time–international normalized ratio, 7.56; and activated partial thromboplastin time, 68.6 s. A chest radiograph showed a cardiothoracic ratio of 68% and mild congestion; moreover, electrocardiography revealed complete atrioventricular (AV) block (Fig. 1). Computed tomography revealed periannular cavity formation. The cavity corresponded to the position of the right coronary cusp of the aortic valve and encroached upon the ventricular septum toward the right ventricle (Fig. 2). A pseudoaneurysm was observed at the proximal anastomotic line following aortic arch replacement (Fig. 2). Transesophageal echocardiography revealed severe regurgitation around the prosthetic valve in the aortic valve position (Fig. 3) and movement of the valve seat. Based on these examinations, the patient was diagnosed with infective prosthetic valve endocarditis, severe aortic regurgitation, periannular abscess, and anastomotic site pseudoaneurysm.

During hospitalization, the patient’s heart failure progressed rapidly. On hospital day 4, he suddenly developed worsening hypoxemia, with the PaO2/FiO2 ratio deteriorating from approximately 300 to 150, accompanied by an increased respiratory rate and the onset of dyspnea at rest. Chest radiography demonstrated progression of bilateral pulmonary congestion. Noninvasive positive-pressure ventilation using bilevel positive airway pressure was required to maintain adequate oxygenation. Accordingly, the patient’s clinical status was categorized as New York Heart Association (NYHA) Class IV due to rapidly progressive heart failure. Preoperative risk assessment indicated an exceptionally high surgical risk, with a EuroSCORE II of 48.23% and a Society of Thoracic Surgeons (STS) score of 26.3%.

No bacteria were identified in the blood cultures. After admission, antibacterial therapy with meropenem and vancomycin was initiated; however, due to acute worsening heart failure, urgent surgery was performed on hospital day five.

**Fig. 4:**
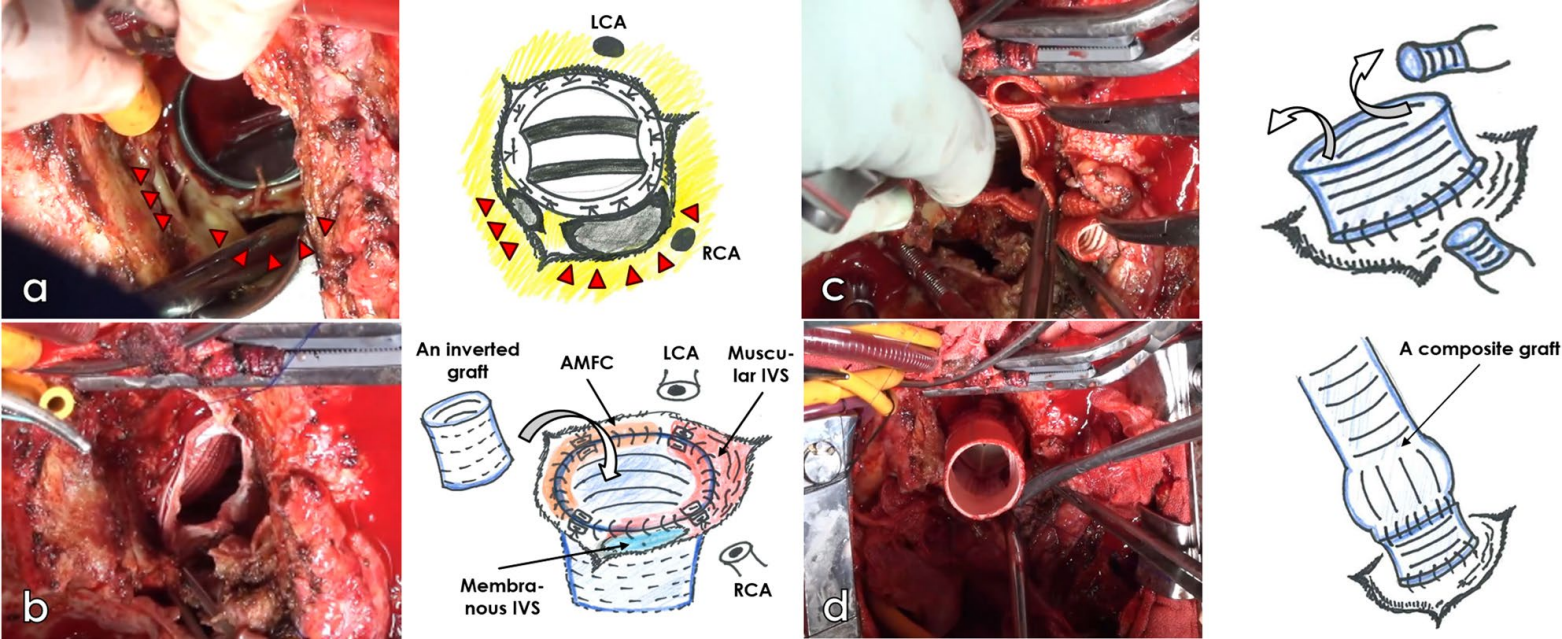
Intraoperative photos and schemas **a** Aortic prosthetic valve detached from the annulus, above which a defect penetrating the right ventricle (red arrowhead) is shown. **b** After complete debridement of the left ventricular outflow tract, an inverted graft about 3 cm in length was inserted into the outflow tract. Edges of the left ventricular outflow tract and the inserted graft were secured with four-point mattress sutures and over-and-over sutures. **c** The inserted tube graft was pulled out of the left ventricular outflow tract. Interposition with short artificial grafts was required to reconstruct the coronary arteries. **d** The composite graft was sutured onto the graft anastomosed to the left ventricular outflow tract *LCA* left coronary artery, *RCA* right coronary artery, *AMFC* Aortomitral fibrous curtain, *Muscular IVS* muscular interventricular septum, *Membranous IVS* membranous interventricular septum

During surgery, the ascending aorta graft was clamped and incised. Cardiac arrest was then induced by delivering antegrade cardioplegia directly into the coronary ostia. Upon examination, partial disruption in the proximal anastomotic line of the artificial graft was observed. Furthermore, the aortic prosthetic valve was detached from the annulus, which extends from the right coronary cusp to the non-coronary cusp. A tissue defect in the ventricular septum was also noted (Fig. 4a). After removing the prosthetic valve and resecting the aortic root tissue while preserving the coronary artery ostia, a periannular abscess was found to have formed from the right coronary cusp to the non-coronary cusp. The abscess encroached upon the ventricular septal muscle and perforated into the right ventricle, near the commissure of the septal and anterior tricuspid valve leaflets. Using sharp curettes, we meticulously eradicated the abscess. We then closed the perforation by direct suturing from the right ventricular side.

The infected tissue was completely cleared from the left ventricular outflow tract (LVOT). However, the anterior part of the aortic annulus was lost, which interrupted continuity with the LV-Ao. We selected a Gelweave Valsalva graft (diameter, 28 mm; Vascutek, Renfrew, UK) to match the size of the LVOT. We cut the body of the graft to 3 cm, inverted it, and inserted it into the LVOT. Four horizontal mattress sutures were placed from the inside of the inserted graft to the epicardial side, fully catching the interventricular septum below the periannular cavity and aortomitral fibrous curtain (AMFC). The edges of the inserted graft and LVOT were sutured with an over-and-over suture (Fig. 4b). The inserted tube graft was pulled out from the LVOT and shortened, leaving 1 cm of length (Fig. 4c). A composite graft (J-graft Valsalva 28 mm and Inspiris Resilia bioprosthesis 23 mm [Edwards Lifesciences Corporation, Irvine, CA, USA]) was sutured onto the graft anastomosed to the LVOT (Fig. 4d). The coronary arteries were reconstructed using short artificial grafts. Even after LVOT reconstruction, there was no uncontrollable bleeding’; the surgery was safely completed.

No bacteria were identified in tissue cultures. Postoperatively, meropenem and vancomycin were administered for one month, after which meropenem was switched to ceftriaxone and continued throughout hospitalization. A permanent pacemaker was implanted due to complete AV block; the patient was discharged on postoperative day 54. At 1-year follow-up, he remained free of recurrent infection.

## Discussion and conclusions

Surgery for infective endocarditis requires thorough debridement and firm fixation of prosthetic valves and allografts in healthy, uninfected tissue [6]. Therefore, the surgical technique depends on the extent of disruption in the aortic annulus and surrounding tissue. If this disruption remains minimal, pericardial patch reconstruction and aortic valve replacement are the preferred treatments [7]. However, if severe disruption in the aortic annulus and surrounding tissue is noted, then complex aortic root reconstruction is required [8]. Surgery for infective prosthetic valve endocarditis complicated by LV-Ao discontinuity requires aortic root reconstruction with LVOT reconstruction with pericardial and artificial graft to restore LV-Ao continuity. Overall, this is a difficult technique [9].

Proximal anastomotic bleeding is the most common complication of repeated surgery on the aortic root; the incidence of massive bleeding requiring repeat surgery is 13% [7]. In this case, we opted for aortic root replacement using the GIT [5, 10, 11]. With this technique, anastomosis is performed by fully inverting the artificial graft and inserting it into the LVOT, ensuring reliable double suture placement, a good surgical field, and sufficient space to work, thereby achieving excellent hemostatic outcomes [11].

In the present case, the decision to use GIT was dictated by several anatomical, clinical, and logistical factors that made alternative procedures unsuitable. The infection extensively involved the anterior portion of the aortic annulus, extending from the right coronary cusp to the non-coronary cusp, with invasion into the muscular interventricular septum and perforation into the right ventricle. Furthermore, this patient required urgent surgery on hospital day 5 due to rapidly progressive heart failure. The extremely limited preparation time, combined with the redo setting after prior hemiarch replacement, significantly narrowed the range of feasible surgical options.

To clarify the decision-making in this emergency redo setting, Table 1 summarizes the major alternative surgical strategies for prosthetic valve endocarditis with LV–Ao discontinuity, their key requirements, and the case-specific reasons they were not selected. Although Uchida et al. described a reconstructive technique using a xenopericardial patch [12], it required a relatively intact sinus of the Valsalva wall for supra-annular fixation. In our patient, the previous hemiarch replacement and the presence of a pseudoaneurysm at the proximal anastomosis rendered the surrounding tissue too fragile, and infection extended to the sinus of Valsalva. Therefore, simple patch fixation was insufficient. Furthermore, although aortic valve allografts are often considered the gold standard for infection resistance [13], their availability for emergency surgery in Japan is extremely limited. Even in large international series, allografts were used in only 7.5% of root abscess cases [9], reflecting their practical scarcity. The Ross procedure is highly suitable for aortic root reconstruction following extensive debridement and demonstrates long-term survival and low thromboembolic risk [14]. However, this technique was also deemed inappropriate due to its excessive technical complexity and the added operative burden of right ventricular outflow tract reconstruction [4] in a hemodynamically unstable, emergency redo-arch setting.

**Table 1:** Comparative assessment of surgical options for prosthetic aortic valve endocarditis with LV–Ao discontinuity and case-specific reasons for exclusion

Option	Advantages	Key requirements	Why not suitable in this case
Allograft	Superior infection resistance	Immediate availability and precise sizing	Impractical: Extremely limited availability in Japan for emergency salvage
Ross	High durability; Superior infection resistance; Low thromboembolic risk	High technical skill; time-consuming RVOT reconstruction	Unsuitable: Excessive complexity and technical burden of RVOT
Patch + AVR	Minimal prosthetic material; Technically less demanding and simpler alternative	Requires relatively intact sinus of Valsalva wall for supra-annular fixation	Impossible: Extensive annular destruction involving the Valsalva wall and interventricular septum
Rifampicin-soaked graft	Enhanced resistance to Staphylococcus species	Institutional protocol and ethical approval	Unavailable: Institutional ethical approval not yet obtained.
GIT + composite graft	Secure subannular fixation and reliable hemostasis	Acceptance of potential AV block and prosthetic infection risks	Best feasible approach: Most feasible salvage option providing reproducible LVOT reconstruction

Table 1 compares alternative surgical options for prosthetic aortic valve endocarditis with LV–Ao discontinuity, highlighting their requirements and the case-specific reasons they were not selected. The table underscores why GIT with a composite graft was considered the most feasible salvage strategy in this emergency redo setting such as our case

*AVR* aortic valve replacement, *LVOT* left ventricular outflow tract, *LV–Ao* left ventricular–aortic, *RVOT* right ventricular outflow tract, *AV block* atrioventricular block, *GIT* graft insertion technique

Within the framework of the graft insertion technique, the use of a rifampicin-soaked graft could also have been considered, as rifampicin exhibits high bactericidal activity against Staphylococcus species [15]; its combination with systemic vancomycin is recommended to enhance graft resistance against methicillin-resistant Staphylococcus epidermidis [16]. However, this option was not available at our institution at the time of surgery, as the ethical approval for using a rifampicin-soaked graft had not yet been obtained.

Consequently, GIT was selected not merely as one option, but as the most feasible and strategically superior strategy for achieving secure hemostasis and reliable reconstruction.

A potential drawback of this technique is postoperative complete AV block. The rate of pacemaker insertion due to postoperative complete AV block is 0%–18% [5, 11]. This may be unavoidable when extensive debridement is necessary and the inserted artificial graft is firmly fixed to the membranous septum and ventricular septum beneath it [5, 11]. Conversely, Nakamura et al. [11] reported no postoperative AV block in six cases in which mattress sutures were placed at a comparatively high level in the membranous septum. Our patient had complete AV block as a complication prior to surgery; the infection might have reached the conduction system. Furthermore, the extensive destruction of the aortic root left no viable annulus for standard reconstruction after debridement. Thus, we achieved reliable hemostasis and structural stability by anchoring the graft into the muscular septum below the membranous septum and the AMFC, which were the only remaining tissues strong enough to support the reconstruction.

Another potential limitation of this technique is the use of prosthetic interposition grafts to the coronary arteries [5]. Therefore, there is a risk of coronary artery occlusion due to thrombus formation, making using the interposition grafts as short as possible without twisting essential.

Kouchoukos et al. [5] reported 23 cases treated with this surgical technique, achieving a 1- and 5-year survival rates of 86.7% and 82.2%, respectively. Only two patients required reoperation, one patient at 51 months postoperatively for a pseudoaneurysm at an aortic graft-to-graft suture line and the other at 81 months postoperatively for degeneration of a freestyle aortic root graft. Recently, Narita et al. [17] have presented midterm outcomes specifically focusing on GIT for redo aortic root surgery in 14 high-risk patients, with a mean EuroSCORE II of 28.8% and a mean NYHA class Ⅱ. Their study demonstrated a survival rate of 53.1% at one year, which remained constant at 3 and 5 years, without recurrent endocarditis or adverse aortic events on follow-up computed tomography. Considering the postoperative life-threatening condition, these outcomes are favorable [16].

Due to the urgent nature of this case, preparation time was minimal. In line with this scenario, we used a GIT, a straightforward option suitable for emergencies. Under these circumstances, the GIT was selected as the most feasible strategy for achieving secure fixation and reliable hemostasis in this emergency redo setting with limited reconstructive options. This technique allowed secure double-layer fixation—combining deep subannular horizontal mattress sutures anchored in healthy myocardium with reinforcing continuous sutures—thereby ensuring reliable hemostasis even in fragile infected redo tissue. Importantly, the GIT enabled reproducible LVOT reconstruction without requiring specialized allografts or extensive preoperative preparation, which was essential given the extreme urgency of the operation on the fifth hospital day.

Although the GIT itself has been described previously, this case extends its clinical applicability as a vital salvage strategy in an exceptionally high-risk scenario. The novelty of this report is highlighted by the patient’s extreme preoperative risk profile, characterized by a EuroSCORE II of 48.23%, an STS score of 26.3%, and NYHA Class IV status. This predicted mortality risk is considered higher than that previously reported cases. Furthermore, the anatomical complexity—emergency redo surgery for prosthetic valve endocarditis complicated by Ao-LV discontinuity and right ventricular penetration following prior hemiarch replacement—represented very severe conditions. The successful outcome in this case demonstrated that the GIT might be a robust and technically straightforward option even when surgical decision-making is severely constrained by extreme urgency, profound tissue destruction, and limited institutional resource availability.

## Data Availability

Not applicable.

## Abbreviations

AMFC: Aortomitral fibrous curtain
AV: atrioventricular
GIT: graft insertion technique
LV–Ao: left ventricular–aortic
LVOT: left ventricular outflow tract
NYHA: New York Heart Association
STS: Society of Thoracic Surgeons
